# Smoking and Pregnancy — A Review on the First Major Environmental Risk Factor of the Unborn

**DOI:** 10.3390/ijerph10126485

**Published:** 2013-11-29

**Authors:** Mathias Mund, Frank Louwen, Doris Klingelhoefer, Alexander Gerber

**Affiliations:** 1 Institute of Occupational Medicine, Social Medicine and Environmental Medicine, Goethe University Frankfurt am Main, Theodor-Stern-Kai 7, 60590 Frankfurt, Germany; E-Mails: m-coder@hotmail.com (M.M.); gerber@med.uni-frankfurt.de (A.G.); 2 Department of Gynecology and Obstetrics, Goethe University Frankfurt am Main, Theodor-Stern-Kai 7, 60590 Frankfurt, Germany; E-Mail: louwen@em.uni-frankfurt.de

**Keywords:** smoking, pregnancy, pregnant, tobacco, cigarette, prevention, therapy

## Abstract

Smoking cigarettes throughout pregnancy is one of the single most important avoidable causes of adverse pregnancy outcomes and it represents the first major environmental risk of the unborn. If compared with other risk factors in the perinatal period, exposure to tobacco smoke is considered to be amongst the most harmful and it is associated with high rates of long and short term morbidity and mortality for mother and child. A variety of adverse pregnancy outcomes are linked with cigarette consumption before and during pregnancy. Maternal prenatal cigarette smoke disturbs the equilibrium among the oxidant and antioxidant system, has negative impact on the genetic and cellular level of both mother and fetus and causes a large quantity of diseases in the unborn child. These smoking-induced damages for the unborn offspring manifest themselves at various times in life and for most only a very limited range of causal treatment exists. Education, support and assistance are of high importance to decrease maternal and fetal morbidity and mortality, as there are few other avoidable factors which influence a child’s health that profoundly throughout its life. It is imperative that smoking control should be seen as a public health priority.

## 1. Introduction

The fact that smoking cigarettes throughout pregnancy is one of the single most important avoidable cause of adverse pregnancy outcomes, resulting in severe short- and long-term negative effects for the mother and the unborn child has been proven by many different studies [1,2,3,4]. It can be regarded as the first major environmental risk factor that can be encountered by the unborn in the developed and undeveloped world. If compared with other risk factors in the perinatal period, exposure to tobacco smoke is considered to be amongst the most harmful. The byproducts of combustion are believed to inflict more damage on the fetus than the nicotine itself, but due to the complexity and number of dangerous substances it is unknown which toxic effect is caused by exactly which product [5]. This is especially significant as the majority of the smoking-induced harm for the unborn fetus is permanent. Even today modern medicine offers very little or no therapeutic treatments for the long-term negative consequences of being exposed to smoke in-utero [6]. According to a 2010 study from the USA, one of the most significant behavior changes a future mother can make is the complete cessation of smoking in pregnancy, with numerous health benefits for both the woman and her offspring [7].

## 2. Epidemiology and Risk Factors

**Figure 1:**
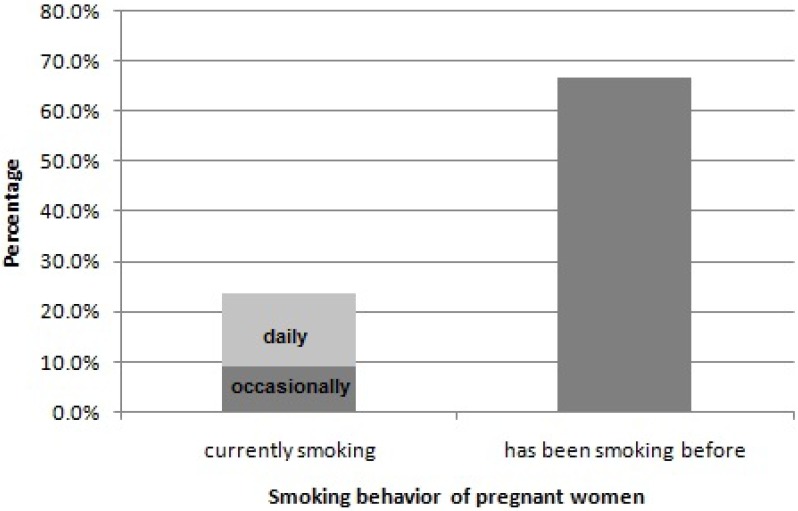
Written information on smoking behavior of 310 pregnant women in Berlin 2001–2002; with kind permission and modified after [4].

**Figure 2:**
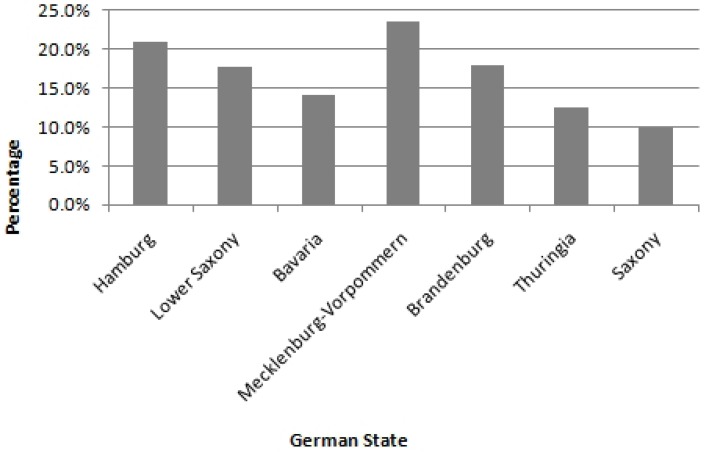
Smoking behavior of pregnant women in different German states 1998–2000; with kind permission and modified after [5]*.*

In 1990 in Germany, a multicenter allergy study surveying 5,395 postpartum women concluded that 28.6% smoked during pregnancy [8]. These results are supported by a second German study from 2001–2002 investigating the cotinine-concentration in the urine of 323 pregnant women in the second trimester. Approximately one quarter of German future mothers smoked during pregnancy (Figure 1) [4], although the rate of smokers varies from state to state. From 1998–2000, the highest frequencies in Germany were observed in the states of Mecklenburg-Vorpommern and Hamburg, whereas the lowest rates were found in Saxony, Thuringia and Bavaria (Figure 2) [9]. In the first years of the new millennium the rate of smoking pregnant women in Germany declined further to approximately 20% [10].

Indicators of socio-economic status are an independent, reliable correlate of active smoking during pregnancy. Studies from Australia and Texas, USA indicated that low socio-economic status three or more times below the poverty line combined with the absence of health insurance increase the risk for smoking during pregnancy, regardless of ethnicity. The smoking rate among Australian women with low socio-economic position, both Aboriginal and non-Aboriginal, was on both occasions approximately two and a half times that of high socio-economic status Aboriginal and non-Aboriginal women [11,12]. Women exposing their unborn child to tobacco smoke were more likely not to be married to the child’s father [11]. Furthermore, women educated only up to high school standard were generally more likely to smoke during pregnancy [13,14,15]. Women employed in serving-related occupations and food preparation were considerably more likely to continue to smoke during pregnancy. They were at a greater risk for drinking alcohol while being pregnant as well. Additionally, women of this risk group showed tendencies to neglect early prenatal care in comparison to women in different occupational groups (Odds Ratio (OR) 1.8–3.0). Female scientist and managers, businesswomen, female artists and other women with higher education were much less likely to smoke during pregnancy compared to the general population of pregnant women (OR 0.2–0.5) [13].

Ethnic minorities often have an increased risk of smoking due to a variety of reasons. According to a Canadian study from 2011, 92% of pregnant Inuit women from Arctic Quebec smoked cigarettes [16]. Roma women in Eastern Europe were 5.2 times more likely (*p* < 0.01) to continue smoking during pregnancy instead of quitting (OR 0.32; 95% Confidence Interval (CI) 0.14–0.72) [17].

## 3. Decreased Fertility and Pregnancy Complications Due to Smoking

For women of childbearing age, active and passive smoking is linked to reduced fertility [18]. Several studies agree that smoking women were more likely to have an abortion, with the total rate of abortion increasing up to 33% [19,20,21,22]. A Japanese case-cohort analysis from 2001 to 2005 involving 180,855 pregnant women concluded that women smoking during pregnancy had statistically considerably elevated risks for various obstetric complications and their rate for stillbirth was estimated by a British study to be increased by 23% (Table 1) [23,24]. For example, a woman who smokes while being pregnant is more than 50% more likely to expose her unborn child to an infection within the womb in comparison to a non-smoker, as the smoker’s Absolute Risk Reduction (ARR) is 1.67 compared to the non-smoker.

**Table 1:** Pregnancy complications due to smoking; modified after [23,24].

Pregnancy complication	ARR	OR	CI (95%)
Preterm rupture of membrane	1.67		1.43–1.96
Chorioamnionitis	1.65		1.36–2.00
Incompetent cervix	1.63		1.35–1.96
Threatened premature delivery	1.38		1.17–1.64
Placental abruption	1.37		1.10–1.72
Pregnancy-induced hypertension	1.20		1.01–1.41
Stillbirth		1.23	1.09–1.38

Even though in this UK study none of the relations with specific congenital abnormalities were significant by themselves, the overall risk of giving birth to a child with a congenital malformation increased by 13% (OR1.13; 95% Confidence interval (CI) 1.01–1.26) [24].

## 4. Biochemical Changes and Alterations

The placenta is an important source of hormones, pro-oxidant agents and antioxidant enzymes and in a physiological pregnancy this vital organ is able to control lipid peroxidation [10]. Several studies concluded that maternal prenatal cigarette smoking disturbs the equilibrium among the oxidant and antioxidant system, thus causing additional oxidative stress and augmenting lipid peroxidation. Smoking during pregnancy increases the free radical damage to the unborn fetus as well as to the mother [10,25,26].

## 5. Smoking and Intrauterine Growth Retardation

Intrauterine growth retardation of the unborn child is the most important smoking-induced pathology [6]. Two studies from 1999 and 2006 associated maternal smoking with an augmented dose-dependent risk for not only adverse birth outcomes such as small-for-gestational age (SGA) and intra-uterine growth restriction but for preterm birth (Adjusted Odds Ratio (AOR) 1.42; 95% CI 1.27–1.59) for both male and female babies as well [2,27]. These findings are supported by numerous studies, which all concluded that children born to mothers who have smoked during pregnancy had significantly decreased birth weights when compared with offspring of non-smokers [28,29,30,31,32,33,34,35,36]. A Brazilian study about newborns exposed to tobacco smoke throughout pregnancy presented an average decrease in birth weight of 223.4 g (95% CI 156.7–290.0), a decrease in birth length of 0.94 cm (95% CI 0.60–1.28), and a decrease in head circumference of 0.69 cm (95% CI 0.42–0.95) [37]. Quitting smoking may have a greater impact on birth weight than refraining from illegal hard drug use, a study by the Quillen College of Medicine, USA examining 265 infants discovered. Among pregnant women who used hard illicit drugs and did not smoke, the adjusted mean birth weight increased 317 g compared to smokers who did not use any illegal drugs [6].

## 6. Alterations in the Genetic and Cellular Level

Smoking cigarettes has a tremendous negative impact on the genetic and cellular level of not only the mother but of the fetus as well, as numerous studies have proven. If certain genetic predispositions are present, the adverse effects of smoking during pregnancy are multiplied [30,32,38]. Genetic and epigenetic mechanisms in combination with cytogenetic damage are believed by a 2012 study from North Carolina, USA, to play an important role in the pathogenesis of malformations and adverse outcomes associated with smoking and pregnancy. In the study, methylation changes in a set of genes (Cytochrome P450 1A1 (CYP1A1), AHRR and GFI1) were present at birth in offspring whose mothers consumed tobacco during pregnancy. These genes seem to play an important role in the aryl hydrocarbon signaling pathway, which mediates the clearance and detoxification of the poisonous components of tobacco smoke [38].

In a Dutch study from May 2012 investigating the effects of smoking on the maternal immune system by examining first-trimester decidual tissue and peripheral blood, researchers concluded that mothers smoking during pregnancy have an altered local and systemic immune system. Using real-time reverse transcription-polymerase chain reaction, flow cytometry and immune-histochemical investigations, the study indicated that more natural-killer cells and inflammatory macrophages are present locally if the woman smokes cigarettes. According to the study smoking mothers have lower percentages of regulatory T-cells than pregnant women who do not smoke [30].

## 7. Diseases Caused by Smoking during Pregnancy

Cigarettes are legal poisons, which damage not only the health of the mother, but jeopardize the health of the unborn child as well. These smoking-induced damages for the unborn offspring manifest themselves at various times in life, some being clearly visible from birth on, others becoming evident only in the following generation. These diseases have one thing in common: the vast majority of them are permanent and for most only a very limited range of causal treatment exists. For many of these diseases only symptomatic therapeutic treatments are available at best [6].

### 7.1. Heart Diseases and Cardiovascular Diseases

The risk of fetal congenital heart defects has been shown by two studies to be at least partially linked to exposure to maternal smoking in early pregnancy or to be directly linked to maternal smoking during pregnancy for some specific subtypes [39,40]. 

A positive correlation between maternal smoking during pregnancy and the risk for congenital heart defects (Table 2) was observed by a study from Atlanta, GA, USA [41] and backed up by a British meta-analysis, which also detected a significant positive correlation between maternal cigarette smoking and fetal cardiovascular and heart defects (OR 1.09; 95% CI 1.02–1.17) (Table 2) [42]. Twelve of 17 subtypes of fetal congenital heart defects have been proven to be more closely associated to maternal cigarette consumption than the other five subtypes. In the study, these twelve subtypes accounted for 71% of fetal congenital heart defects. Fetal septal heart defects as a group were the highest risk for any subtype (Table 2). The incidence of septal defects as a group, atrial septal defects and atrioventricular-septal defects correlated directly with the number of maternal cigarettes smoked [43].

**Table 2:** Smoking and fetal congenital heart defects; modified after [41,42,43].

Congenital heart defect	RR	OR	CI (95%)	n
Congenital heart defects (total)	1.11	1.09	1.02–1.21	18,282
Septal heart defects	1.44		1.16–1.79	2,977
Secundum-type atrial septal defects		1.36	1.04–1.78	
Right ventricular outflow tract defects		1.32	1.06–1.65	
Pulmonary valve stenosis		1.35	1.05–1.74	
Truncus arteriosus		1.90	1.04–3.45	
Transposition of the great arteries		1.79	1.04–3.10	

### 7.2. Hypertension and Kidney Diseases

Maternal cigarette smoking during pregnancy might also affect fetal kidney development. A dose-dependent relationship between the number of cigarettes consumed during pregnancy and fetal kidney volume was observed by a Dutch prospective cohort study of 1,072 children. Researchers found that maternal smoking of ten cigarettes per day correlated with a decreased fetal combined kidney volume compared to women who smoked less than five cigarettes per day (*p* = 0.002). This could predispose the offspring to the development of kidney disease and hypertension later in adult life [44].

### 7.3. Pulmonary Diseases

A study from Detroit, MI, USA, has shown that smoking and pregnancy is significantly linked with a decrease in pulmonary function in offspring later in life [43]. An important increase in pediatric hospitalization and mortality because of respiratory infections in early childhood independent of both birth weight and gestational age was associated by a retrospective case-control analyses of infants born in Washington State from 1987–2004 with having been exposed to maternal smoking in-utero (AOR 1.69; 95% CI 1.63–1.76) [26].

Studies from the New York University School of Medicine, USA and from the Karolinska Institute, Sweden, found that pregnant women who have smoked cigarettes increased the risk of wheezing and asthma for their children. If children have been exposed to maternal smoking in-utero, but not in the first year after birth, the AOR increased for wheeze and asthma. At the age of four to six years the OR increased to 1.39 for wheeze (95% CI 1.08–1.77) and 1.65 for asthma (95% CI 1.18–2.31). The likeliness to suffer from these two diseases in later childhood increased significantly in relation to the amount of exposure to maternal cigarette smoke in-utero during the first trimester of pregnancy [37,44].

### 7.4. Gastrointestinal Diseases

A Danish follow-up study of singleton infants concluded that maternal smoking during pregnancy increases the risk for infantile colic even after adjustment for factors like maternal age, birth weight, gestational age, breastfeeding and paternal smoking. A two-fold increased risk for infantile colic was observed in offspring of women who smoked at least 15 cigarettes per day during their pregnancy (Table 3) [45]. These findings are consistent with a study from the UK on non-chromosomal birth defects, which discovered that babies of mothers who have smoked during pregnancy were also at higher risk for gastrointestinal defects, gastroschisis and anal atresia (Table 3) [40].

**Table 3:** Maternal smoking and gastrointestinal diseases in the offspring; modified after [42,45].

Gastrointestinal disease	RR	OR	CI (95%)
Gastrointestinal defects		1.27	1.18–1.36
Infantile colic	2.1		1.4–3.2
Gastroschisis		1.5	1.28–1.76
Anal atresia		1.2	1.06–1.36

## 8. BMI and Obesity

Multiple studies agree that maternal smoking during pregnancy harms linear growth, promotes increased Body Mass Index (BMI) in children and augments the risk for obesity in childhood and adult life [46,47,48,49]. Exposure to cigarettes in-utero causes increased mean BMI, pulse rate, waist circumference and waist-hip-ratio. Offspring of mothers who smoked at other times in the child’s life but not during their pregnancy have similar mean risk factors compared to children whose mothers never smoked. Among children of mothers who smoked during pregnancy, the degree of overweight children with an increased BMI is positively correlated with the duration of the maternal smoking, which is due to reduced height and increased amount of body fat [47,49,50,51].

## 9. Alterations in Neurology and Psychological Behavior

Early child neurodevelopment plays a key role in the potential of preserving human intelligence and health in the next generation, as stated in a study from the University of Iowa, USA [52]. Similarly, a review from Providence, RI, USA concluded that the various adverse aspects of long-term in-utero exposure to active and passive smoking on the neurological development of the child and its behavior have become the focus of a few investigations in recent years [53].

Maternal smoking during pregnancy has been linked to growth restriction and decrease in the size of the fetal brain by numerous studies. It has been proven that the density of important parts of the fetal brain, namely the cerebellum and the corpus callosum is diminished. A decrease in coordination within the different parts of the fetal brain during processing of information and a deceleration in the ability to adequately respond to external stimuli and subtly diminished motor competence predominantly on the non-dominant side have been shown by these studies [53,54,55].

In a 2011 Finish cohort study investigating various cognitive functions such as general reasoning, visual-motor integration, verbal competence and language comprehension in 1,019 infants, researchers found a pattern between heavy cigarette consumption prior to pregnancy with poorer cognitive executive function proficiency in the offspring. Interestingly, the results indicated a poorer performance of the offspring even if the woman had ceased smoking before conception. Children of mothers who smoked more than ten cigarettes per day before pregnancy but none during pregnancy scored 12.07 (95% CI 4.07–20.08) age-standardized points less in general reasoning and 11.23 (95% CI 2.81–19.66) age-standardized points less in language comprehension tests compared to children of mothers who never smoked [56]. Independent of maternal education levels offspring born to smoking mothers were also more probable to achieve less in math (OR 2.78; 95% CI 1.59–4.87) and reading (OR 2.00; 95% CI 1.10–3.63) compared to children of non-smokers [57].

## 10. Addiction

Pregnancy functions as a motivator to quit smoking and is a good time to stop smoking since pregnant women are more probable to be in an advanced phase of behavioral change. This was proven by a study from Philadelphia, PA, USA to be true even for pregnant nicotine- and opioid-dependent patients in substance abuse programs [58]. In a comparative study by the University of Padua, Italy pregnant women reported decreased levels of nicotine use and lower level of self-reported cigarette cravings reaching a statistically significant level in comparison to non-pregnant female patients [59].

Generally a low uptake of smoking cessation programs among pregnant females has been observed by many different studies. Pregnant females with high levels of nicotine dependence and several occupational groups such as women in serving-related occupations and in food preparation were the most unlikely socio-economic group to give up smoking during pregnancy or to take advantage of smoking cessation interventions [13,17,60,61]. In particular, smoking cessation programs for pregnant low-income smokers were for the most part unsuccessful, a study from Buffalo, NY, USA discovered [62].

Several studies indicated that even if the pregnant woman has been able to quit or reduce smoking, the smoking rate increased again after giving birth. During pregnancy, 45% of smoking women were able to quit smoking but at 24 weeks post-delivery only 34.6% of women remained abstinent and almost 80% continued to smoke within one year postpartum [3,63,64]. Unfortunately, quitting smoking during pregnancy was proven by a study from Vermont, GA, USA, investigating data from over 40,000 adults, not to be significantly connected to the smoking status three years later [65].

## 11. Therapy and Anti-Smoking Programs

Pregnant women who smoke welcome receiving advice on how to quit or reduce smoking from midwives, a comparative study from the UK discovered. Nevertheless, according to the study, they tend to have negative expectations of smoking cessation programs services, even though the experiences of those who have participated are positive [66]. A South African study on midwives concluded that the way in which medical personal communicate about the issue of smoking and pregnancy is of high importance. The most positive response from pregnant women was obtained by the patient-centered approach which is based on short motivational interviewing and a trusting and cooperative connection between midwife and patient. Medical personal practicing this modern approach was more successful in fulfilling their function in smoking cessation programs [67].

A comparative study from Italy recommended that smoking cessation campaigns should not only target the smoking future mother. Instead, the social network including partners, roommates, family and friends who smoke should be included as well, especially in postpartum women [59]. A systematic literature review from Vancouver, BC, Canada attributed a significant role to the male partner’s smoking behavior. Their support for the woman’s efforts to diminish or abstain from smoking cigarettes may impact her success in doing so. Regardless of the significance of partner smoking, there are only a small number of effective anti-smoking programs for pregnant and postpartum females that take male partners into consideration [68].

In comparison to smoking pregnant women who received only brief routine advice to quit, a US randomized controlled trial discovered that women who were additionally educated by a “Commit to Quit” video, a “Pregnant Woman’s Guide to Quit Smoking” and counseling achieved a significantly higher cessation rate (17.3% *vs.* 8.8%) [69].

Unconventional methods can be effective if additionally added to established methods of treatment. A small Japanese study (*n* = 48) investigating the effectiveness of an e-learning program which supports pregnant women willing to quit smoking by use of a cell phone internet connection reported a high achievement rate of 71.1%. The maternal carbon monoxide exhalation levels were significantly reduced from 6.43 (±4.5) ppm to 0.29 (±1.08) after three months (*p* < 0.001) [70]. These findings certainly have to be regarded in the context of a technologically highly advanced society, but the very principle should be applicable to a country of the developing world as well.

Two studies from Australia and Italy indicated that in addition to being significantly less likely to smoke cigarettes in general (AOR 0.10; 95% CI 0.02–0.68), women breastfeeding their children were in the short and long term more likely to stay abstinent or smoke less compared to non-breastfeeding women. Breastfeeding in itself may also indirectly support smoking cessation, even without the presence of specific anti-smoking campaigns and should therefore be widely promoted [71,72].

Additionally to high quality educational programs the pharmacological aspect needs to be taken more into consideration [73]. A Canadian study from 2012 emphasized on the importance of using suitable drugs which include nicotine in different forms as replacement therapy and sustained-release bupropion. In the study, nicotine replacement therapy and bupropion did not seem to augment the prenatal risk of malformations. For an additional drug varenicline, insufficient data has been collected to safely advise its use during pregnancy. Taking into consideration that these pharmacological agents on their own are only marginally successful in smoking cessation, their prescription should at all times be combined with behavioral counseling and education to optimize success rates [74].

## 12. Conclusions

The epidemiology, pathogenesis and methods of education and prevention of smoking and pregnancy are of great interest and importance for public health, as there are few other avoidable factors which influence a child’s health that profoundly throughout its life. In 1990 in Germany, 28.6% of women smoke during pregnancy; the highest frequencies are observed in Mecklenburg-Vorpommern and Hamburg, the lowest in Saxony, Thuringia and Bavaria. In the first years of the new millennium the rate declines to approximately 20%. Low socio-economic status, lower education and belonging to an ethnic minority increase the risk for smoking during pregnancy significantly.

Smoking women are up to 33% more likely to have an abortion and suffer from considerably elevated risks for various obstetric complications. For smokers the rate for stillbirth is increased by 23% and the overall risk of giving birth to a child with a congenital malformation increases by 13%. Babies of smokers are more likely to be SGA and suffer from intra-uterine growth restriction as well as to be born before term.

Smoking cigarettes has a negative impact on the maternal and fetal genetic and cellular level; the increase in fetal septal heart defects correlate directly with the number of maternal cigarettes smoked during pregnancy. Maternal cigarette smoking during pregnancy is likely to affect fetal kidney development leading to kidney disease and hypertension later in adult life. Smoking and pregnancy is significantly linked with a decrease in pulmonary function in addition to wheezing, asthma and respiratory infections in offspring later in life. Additionally, an elevated risk for various gastrointestinal defects is observed in offspring of smokers. Smoking during pregnancy harms linear growth, promotes increased BMI in children and augments the risk for obesity in childhood and adult life. Maternal smoking during pregnancy has been linked to decrease in the size of the fetal brain as well as to diminish general reasoning, visual-motor integration, verbal competence and language comprehension in the offspring.

During pregnancy, 45% of smoking women are able to quit smoking, but almost 80% continue to smoke within one year postpartum. A low uptake of smoking cessation programs among pregnant females has been observed in particular among pregnant low-income smokers. Pregnant women who smoke welcome receiving advice on how to quit; the way in which medical personal communicate about the issue of smoking and pregnancy is of high importance. The most positive response is obtained by the patient-centered approach based on short motivational interviewing and a trusting and cooperative connection. For these reasons, medical personal should be specially trained in this highly sensitive task. Smoking cessation campaigns should target the smoking future mother as well as her social network. Male partners, roommates, family and friends should be included, especially in postpartum women. The importance of breastfeeding cannot be emphasized enough. Smoking cessation campaigns should combine these conventional methods with modern building blocks like multimedia, videos, computers and e-learning programs in addition to mobile telephone communication. Another focus should be set on the pharmacological aspect with nicotine replacement therapy and other suitable supporting drugs. Culturally and socio-economically sensitive smoking cessation programs need to be established for the ethnic and socio-economic groups of pregnant women most at risk.
